# Supplementary material for: STRyper: A macOS application for microsatellite genotyping and chromatogram management
Source: PLoS One. 2025 Feb 20;20(2):e0318806. doi: 10.1371/journal.pone.0318806 (PMC11841898; doi:10.1371/journal.pone.0318806)
Supplement: S1 Text — This file describes methods for peak delineation, baseline fluorescence level subtraction, determination of crosstalk, size assignment of molecular ladder fragments, detection of microsatellite alleles, and an overview of the database managed by STRyper. (PDF) [file pone.0318806.s001.pdf]

Supporting information for "STRyper: a macOS application for microsatellite genotyping and chromatogram management"

Peak delineation

To delineate peaks in the fluorescence data, STRyper uses a simple method that enumerates fluorescence levels from the first to the last recorded scan. A scan is a data point that is denoted by an integer index varying from 0 to the total number of data points.

The method records the lowest fluorescence level (l) and the highest level (h), and their respective scan numbers (s_l, s_h), observed up to the current scan number (s_f) whose fluorescence level is denoted as f. A peak is delineated if $h > t$, $l/h \leq r$ and $f/h \leq r$, t being the minimal fluorescence level to consider a peak (by default, 100 fluorescence units) and r being a parameter denoting the minimum peak elevation above the background. Horizontally, the peak starts at scan s_l, and its tip is at scan s_h. Its right boundary will correspond to the left boundary of the next peak. This method thus generates contiguous peaks.

For best results, it was found that three rounds of peak detection should be applied to the data, each round being followed by one pass of baseline fluorescence level subtraction (see next section). The first two rounds use a value of 0.7 for r, a modest peak elevation that allows the detection of faint peaks. The last iteration uses r = 0.5, which means that a peak must be at least twice higher than the background level, considering that baseline fluorescence level subtraction makes peak stand-out more.

After these three rounds, the left and right boundaries of each peak are delineated by the closest scan from each side of the peak's tip that has a fluorescence level of 0, using fluorescence levels with baseline level subtracted. This produces non-contiguous peaks.

Baseline fluorescence level subtraction

STRyper subtracts the baseline fluorescence level of a trace after peaks are delineated (see previous section) as follows. A virtual line segment is drawn from the start to the end of each peak (Figure S1). For a given scan number s_f, the height of the segment is denoted as y and is considered the "baseline fluorescence". The recorded fluorescence level for the scan is denoted as f and the fluorescence level at the peak tip is denoted as h.

For each value of s_f within the peak, a value v to subtract to the fluorescence level depends on whether the absolute height of peaks should be preserved. If so, $v = y(h - f)/(h - y)$. Otherwise, $v = y$. If v is negative, it is set to 0. The new value for the fluorescence level is $f - v$. After this operation, each peak starts and ends at a fluorescence level of 0.

The same operation is performed between peaks to reduce the background noise. Between peaks, $v = y$.

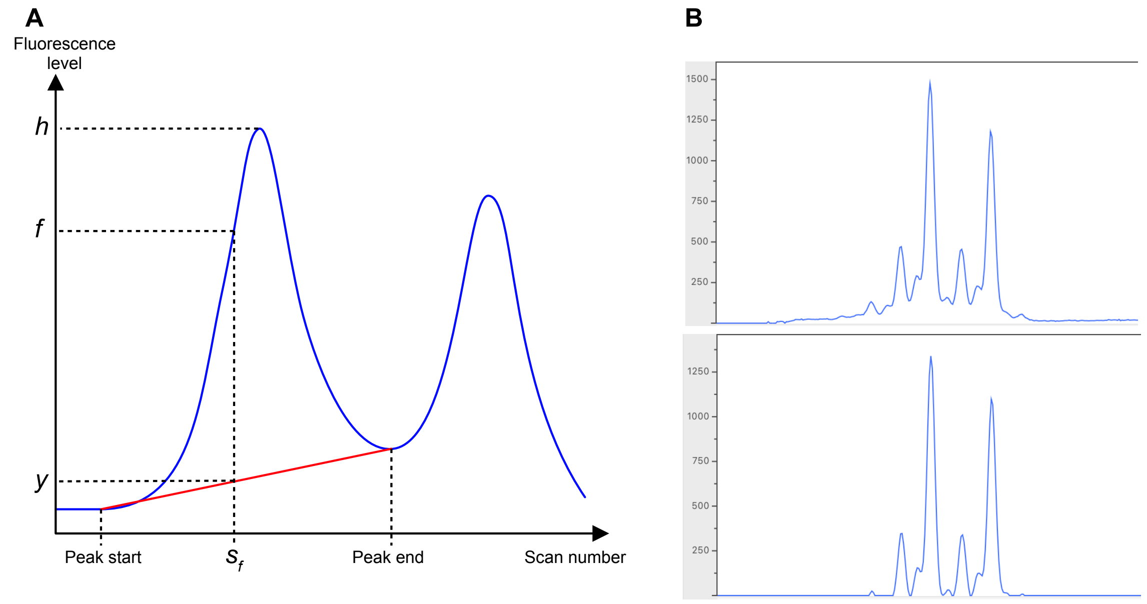

Figure S1. Subtraction of baseline fluorescence level. A) Principle of the method. Symbols are defined in the supplementary text. B) Effect of the three passes of the method (see supplementary text) on fluorescence curves. Top: raw fluorescence data. Bottom: fluorescence data after baseline fluorescence level was subtracted.

Determination of crosstalk

STRyper determines whether a peak in fluorescence results from interference between channels, i.e., crosstalk. This inference relies on the presence of saturation, or of higher peak of similar shapes, in other channels.

A chromatogram file lists each scan number for which the signal saturated the sequencer camera but does not specify which channel caused the saturation. STRyper determines this channel by first delineating regions composed of consecutive scan numbers where saturation occurred.

For each region, the channel that is considered to have caused saturation is the one whose fluorescence level is the highest at the first scan of the region. This criterion does not compare maximum/average fluorescence levels over the region between channels, because the peak at the channel that caused saturation if often clipped and may be smaller than peaks of other channels in the region. However, this peak has the highest fluorescence level at the point where saturation began.

A peak is considered to results from crosstalk if the following conditions are met: (i) its tip lies within a region where saturation is caused by another channel, and (ii) the fluorescence level at the peak tip is at least twice those recorded at the scan preceding the start and the scan after the end of the region. Criterion (ii) accounts for the fact that several DNA fragment may have migrated at the same speed, such that legit peaks appear at the same locations. However, the fluorescence level at a peak resulting from crosstalk should not be high before the saturation from another channel is recorded.

Alternatively, crosstalk may cause a "crater" in other channels, that is, sharp peaks at the edges of the saturated region. If a small peak lies near such edge and sharply decreases within the saturated region, the peak is considered to result from crosstalk.

If a focus peak is not considered are resulting from crosstalk based on these criteria, the program inspects other channels to find the one with highest fluorescence level at the peak tip, and for which the fluorescence level is at least 1.6 times that at the peak tip. If it finds one, it then evaluates how much peaks of both channels overlap, using two criteria. The program first scales down the higher peak such that is elevation corresponds to the smaller. It then measures the peak areas by summing fluorescence levels. The first criterion is considered passed if the area representing the intersection between peaks is at least 30% of the area representing the union of the peaks. The second criterion precisely evaluates how much the peak horizontal positions are aligned. For that, the difference in fluorescence level (curve height) between channels is computed at each scan along the range encompassing both peaks. The sign of the difference is reversed if the scan is greater than the scan of a given peak's tip. For each peak, these differences are summed across all scans of the range. The second criterion is considered passed if the absolute value of each sum is less than 30% the combined areas of the peaks. If both criteria are met, the program checks if other peaks in the channel that may have induced crosstalk also induced crosstalk in the focus channel.

This inspection relies on the expected ratio of peak heights between the two channels, which should be rather constant in the case of crosstalk and in the absence of saturation. If another peak does not appear to have induced crosstalk, then the peak under consideration is not considered to result from crosstalk.

Size assignment of molecular ladder fragments

The algorithm conceived to assign sizes to molecular ladder fragments inspects peak in the appropriate channel, ignoring those resulting from crosstalk (see previous section). In the following, the “scan number” of a peak refers to the scan at its tip.

Peaks are first enumerated by decreasing scan numbers, and the average peak height is computed at each step. Any peaks whose height is at least twice the current average and whose scan number is less than 1/3 total number of scans in the trace is discarded. This eliminates high-intensity peaks of short size (in base pairs) resulting from degradation of the molecular ladder.

The algorithm then discards weak peaks amounting to “noise”, which sometimes affect the data. To do so, remaining peaks are enumerated by decreasing height. The enumeration stops when the number of enumerated peaks corresponds to the number of sizes specified in the size standard, or when a peak is at least three times smaller than the previous one. Any peak that is at least twice as small as the least enumerated peak is discarded.

To assign remaining peaks to sizes defined in the size standard, peaks are ordered by increasing scan number. The method assigns the lowest size to the first peak, and the largest size to the last peak. To understand the process, picture a straight line of equation $y = a + bx$ passing through these two peaks on a plot where the x axis represents scan numbers, and the y axis sizes in base pairs.

Peaks are then enumerated in decreasing order, starting from the second-to-last. The size of the fragment causing a peak is estimated as $a + bx$, x being the peak scan number. The size of the size standard that is the closest to the observed size is assigned to the peak, only if the difference between both sizes is less than 15 bp in absolute value.

The next peak is evaluated in the same fashion. If it is assigned to the same size as a previous peak, both peaks are confronted to retain the one whose predicted size is the closest. The a

and b parameter are updated to correspond to the line connecting the two peaks that were assigned last. Hence, the size/scan relationship dynamically changes to account for non-linearity.

At the end of the procedure, the shortest size of the size standard may be assigned to a different peak than the one of lowest scan number. This is not the case for the longest size, which remains assigned to the peak of largest scan number, although this assignment might be erroneous (this is addressed using subsequent iterations, as described below).

A quality index is computed to evaluate the assignments. This index relies on the residuals of the linear regression between scan number and size in base pairs, using ordinary least squares. For each pair of successive points (peaks), the difference between residuals is divided by the difference between scan numbers, both in absolute value. The mean of these ratios is computed. The inverse of this mean, multiplied by the percentage of sizes that were assigned to peaks, constitutes the quality index. If this index is higher than a certain value (chosen at 100), the number of assigned sizes is recorded as a reference.

Further iterations of assignments are performed by decrementing the longest assignable size (to consider the possibility that electrophoresis failed or stopped before the last fragment was detected), then by decrementing the last assignable peak. Assignments are not recorded if the number of assigned sizes is lower than the reference, and iterations stop when the number of assignable sizes/peaks is lower than the reference.

In the end, the set of assignments that yielded the best quality index is retained.

Detection of microsatellite alleles

To identify microsatellite alleles at a marker in a chromatogram, peaks found in the marker range are first sorted by decreasing number of saturated scans they induced, then by decreasing height (fluorescence level). This sorting accounts for clipping due to saturation of the fluorescence signal. Hereafter, a peak position/size (in base pairs) refers to the position of its tip, hence the estimated length of the DNA fragment that induced the peak.

For each peak (hereafter called a “reference” peak), neighboring peaks are successively inspected at increasing distance to identify peak clusters. Neighbors that are at the left (lower scan numbers) are inspected before those at the right. Briefly, the inspection first

evaluates if a neighbor resulted from stuttering: the distance between the neighbor and the reference peak (of from a previously inspected neighbor already considered as a stutter) must be the motive length of the marker ± 0.5 bp. In addition, the neighbor must be smaller than the reference peak or than a previously inspected neighbor already considered as a stutter. If these requirements are met, the neighbor is flagged as a “child” of the reference peak, that is, both are considered part of the same cluster and have arisen from amplification of the same allele. If the distance between a neighbor and the reference peak (or a previously inspected neighbor) lies between 0.5 and 1.5 bp, and if the neighbor is smaller than the reference peak, the neighbor is interpreted as resulting from adenylation of the amplicon, hence as a child peak. This is also the case if the neighbor is distant from the motive length ± 0.5 bp from a previous peak considered as resulting from adenylation.

The application stops the inspection of neighbors (at the left or at the right or a reference peak) if a neighbor is already flagged as a child from a reference peak inspected prior, or if its distance from the last inspected neighbor exceeds the motive length + 0.5 pb, in which case it the neighbor is not considered as a child.

Additional checks based on peak heights are implemented to avoid considering alleles differing by only one repeat motive as part of the same cluster. One check accounts for short allele dominance in heterozygotes: the fact that the longer allele is almost always amplified with lower yield during PCR. The resulting peak is therefore smaller, but it must not be considered as a result a stuttering. To account for dropout, a neighboring peak at the right of the reference peak is considered as resulting from stuttering only if its height is <30% that of the reference peak. Conversely, the method also considers rare cases where the shorter allele has amplified with lower yield than the longer. If allele lengths differ by just one repeat motive, the left peak may be erroneously considered as a stutter. To avoid this, the method computes the ratio of peak heights (left peak / right peak). If the ratio is ≥ 0.7, the program looks for an additional stutter peak at the left, at distance that is the motive length ± 0.5. This check assumes that a first peak arising from stuttering is followed by others with similar height ratio between neighboring peaks. If no such peak is found, the left peak is not considered as a child peak, and the inspection of neighbors that are at the left of the reference peak stops. Note that if the shorter allele amplifies with a much lower yield than

the longer allele, distinguishing it from a stutter would require comparing individuals, which the application does not.

After neighbors are inspected for all reference peaks, all peaks are processed by decreasing height. The first peak is always considered as an allele and any subsequent peak will be as well if the following conditions are fulfilled. First, the number of alleles must not exceed the marker ploidy and the peak must not be a child peak. Then, a ratio is computed by dividing its height with that of the last peak considered as an allele. If the focus peak is at the left of the allele and the ratio is ≥ 0.7, it is considered as another allele. If the subsequent peak is at the right, the ratio must exceed 0.3, or the ratio multiplied by the absolute difference in peak positions (in bp) must exceed 4. This second condition allows alleles that are much longer to yield peaks that are much smaller, accounting for short allele dominance. A peak not considered as an allele is flagged as a “supplementary” peak (see main text) if it is not a child peak and if its height is ≥20% of the height of last peak considered as an allele (or ≥12% of the allele height if the focus peak itself has child peaks).

The method also considers a special case where two alleles of a heterozygote differ by only one nucleotide in length. In this case, one allele may be wrongly considered as a peak resulting from adenylation and a homozygous genotype would be called. Only comparison with other genotypes may indicate whether the degree of adenylation of the marker is compatible with this assessment. Therefore, during alle call, the application records the ratio between the height of a given peak and the height of a peak inferred as an allele, if the peak is within 1.5 bp from the allele. This ratio is called l if the fragment represented by the peak is longer than the allele or s if the fragment is shorter. $L = \max(l)$ and $S = \max(s)$ are computed for a given genotype. In the absence of detectable adenylation, these maxima would be zero. The peak that has the highest ratio is considered as a “possible allele” of the genotype.

If, during the same allele call, at least two genotypes were considered as heterozygous and at least one as homozygous for a given diploid marker, the application computes arithmetic means $M(L)$ and $M(S)$ over all genotypes that are heterozygous. The application then inspects the possible allele of every homozygous genotype. If a possible allele fulfills ($l > 6\times M(L)$ and $l \geq 0.5$) or ($s > 6\times M(S)$ and $s \geq 0.5$), it is promoted as allele.

Overview of the database managed by STRyper

STRyper manages a database composed of objects of different classes that are represented in Figure S2.

An object of class *Marker* describes a microsatellite marker by specifying its *name*, and the *start* and *end* of the range of expected alleles expressed in base pairs (these attributes are inherited from a superclass called *Region*). *motiveLength* specifies the length of the repeat motive, *channel* the channel (wavelength) used to reveal amplicons (blue, green, black/yellow, or red) and *ploidy* is self-explanatory. The set called *bins* points to the bins defined for the marker. The *start* and *end* attributes of a Bin (inherited from the Region class) specify the expected range of an allele. A marker also points to the *Panel* (i.e., the multiplex) it belongs to, which reciprocally points to its markers via a set called *markers*.

An object of the *Chromatogram* class stores data imported from a .fsa or .hid file into various attributes, including the sample name (*sampleName* attribute), the *plate* name, the *well* identifier, the time of the end of sequencer run (*runStopTime*), the number of scans recorded (*nScans*), the indices of scans for which the camera was saturated (*offScaleScans*), etc. The *panel* relationship points to the panel of markers that were amplified to generate the chromatogram. Reciprocally, a panel points to all chromatograms that use it for genotyping, via a set called *samples*.

Via the *traces* set, a chromatogram points to four or five objects of class *Trace*, each of which encodes the raw fluorescence data (in the *rawData* attribute) measured at a *channel*. Peaks identified in each trace are stored in the *peaks* attribute, which is an array of structures composed of three integers: the scan at the start of the peak, the number of scans from the start to the tip, and the number of scans from the tip to the end of the peak. The *dyeName* attribute stores the name of dye that emitted the recorded fluorescence used (e.g., "6-FAM", "LIZ") and *isLadder* tells whether the trace represents the molecular ladder that was added to the sample before electrophoresis.

The size standard defining the molecular ladder is referred to by the chromatogram, via its *sizeStandard* relationship. A SizeStandard object has a *name* (e.g., "GeneScan 500") and a set of *SizeStandardSize* objects (called *sizes*), each of which has a *size* attribute specifying a

fragment size in base pairs. This set facilitates adding, removing or changing sizes. The *editable* attribute of a size standard tells whether its *sizes* can be edited by the user.

The *fragments* relationship of a trace points to *LadderFragment* objects. Such object defines a DNA fragment that produced a peak identified in the trace. Its *scan* attribute refers to the scan at the tip of the peak (hence the location of the fragment in the trace), and its *size* attribute is the size (in base pairs, taken from the size standard) that was attributed by the method described in section “Size assignment of molecular ladder fragments”. The *name* of the fragment (shown to the user) is its *size* encoded as characters. The *offset* attribute is the difference (in base pairs) between the location of the *scan* (computed via the *coefs* attribute of the chromatogram, see below) and the *size*. This helps users detect size assignment errors. The coefficients of the polynomial that was fitted based on detected ladder fragments (see main text section “**Erreur ! Source du renvoi introuvable.**”) are stored as an array of floating-point numbers in the *coefs* attribute of the chromatogram to which the trace belongs.

For a trace that is not a molecular ladder, the *fragments* set can only contain *Allele* objects. An Allele defines a DNA fragment that produced a peak in the range of a marker (from the panel applied to the trace’s chromatogram) that has the same *channel* as the trace. The *size* attribute of an allele is computed from its *scan*, and its *name* is independent of its *size*. The *additional* attribute tells whether the allele represents an additional fragment (see main text section “**Erreur ! Source du renvoi introuvable.**”). An allele does not use the *offset* attribute that it inherits from the LadderFragment class.

A *Genotype* object regroups alleles that were found in an individual (chromatogram) at a marker. It therefore points to these objects using relationships called *alleles*, *sample* and *marker* respectively. A genotype stores the *a* and *b* parameters used to correct for allele sizes (see main text section “**Erreur ! Source du renvoi introuvable.**”) in an attribute called *offset*. Indeed, these offset parameters are specific to an individual analyzed at a marker, hence of a genotype. The genotype *status* attribute tells the user whether the genotype has been called, whether alleles were found, the genotype has been manually edited, etc.

The Folder class lets users organize chromatograms and marker panels. A folder has a name and points to subfolders via a set called subfolders. Reciprocally, each subfolder points to its parent folder via its parent relationship. A nested hierarchy of folders can therefore be

constructed. As a subclass of Folder, a Panel has a name and a parent, which is an object of the PanelFolder class. The subfolders of such object may be panels and/or other panel folders. As a Folder, a Panel can technically have subfolders, but the application code prevents this.

A SampleFolder is a Folder that can contains Chromatogram objects in a set called samples. A SmartFolder specifies a search predicate in its *searchPredicate* attribute. When it is accessed, a smart folder returns chromatogram objects found across the whole database via the search predicate. A smart folder has a parent that is the same for all smart folders, it but is not allowed to contain subfolders.

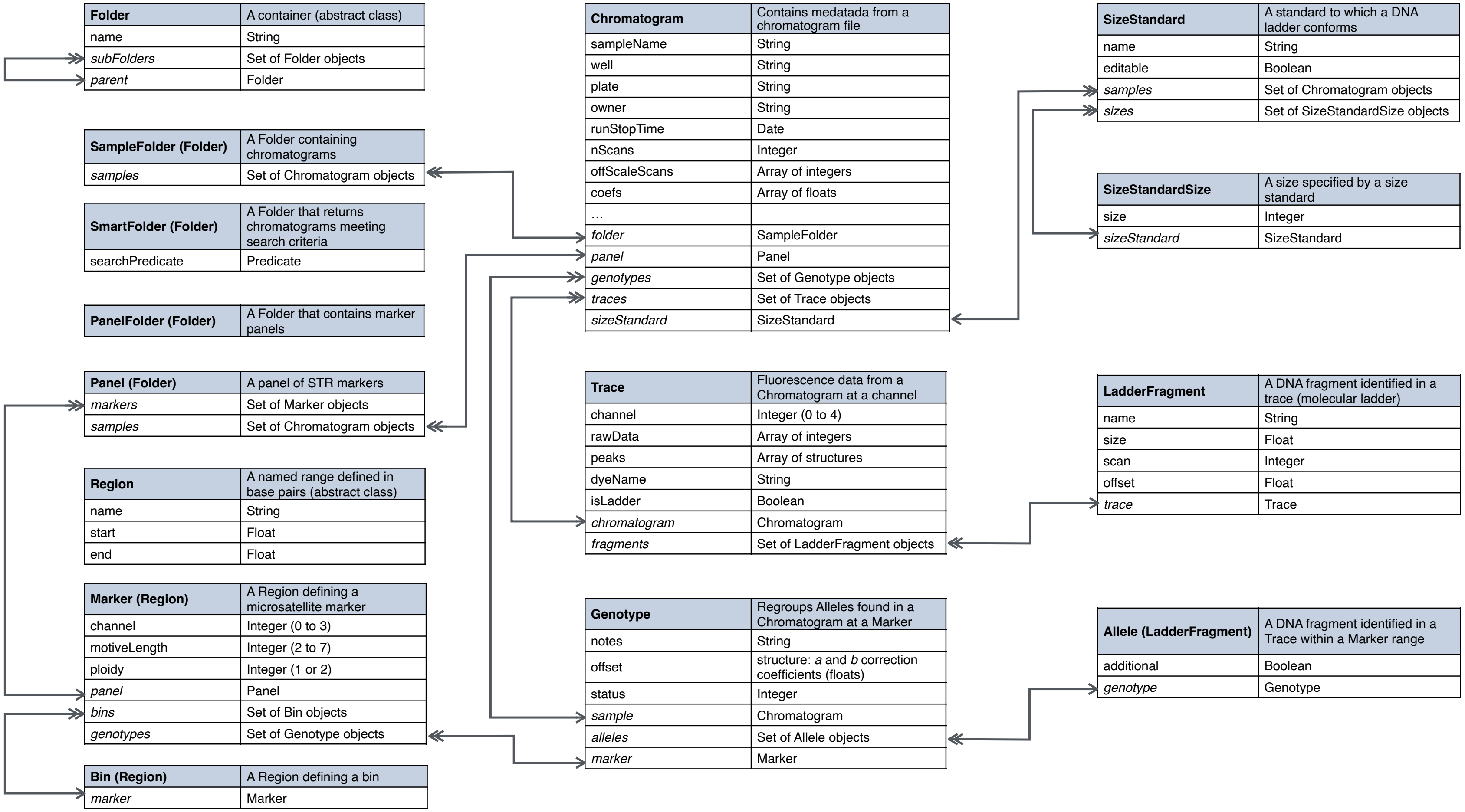

Figure S2. Overview of the database managed by STRyper. Each table describes a class defined in the application. The top left cell shows the class name followed by the name of its superclass in parentheses, if relevant. The top-right cell shows a brief description of the class. Below the table header, the left column lists the names of attributes and relationships of the class. Relationship names are italicized. The right column specifies the type of each attribute/relationship. A class inherits attributes and relationships from its superclass, but it may not use them in the application code. All relationships are reciprocal, and reciprocity is represented by arrows. Single arrows point to to-one relationships (pointers to a single object) and double arrows point to to-

many relationships (sets of pointers to several objects). Only attributes and relationships saved in the database are shown. For a complete definition of these classes, see header files at https://github.com/jeanlain/STRyper/tree/main/STRyper/Entities/
