# Supplementary material for: STRyper: A macOS application for microsatellite genotyping and chromatogram management
Source: PLoS One. 2025 Feb 20;20(2):e0318806. doi: 10.1371/journal.pone.0318806 (PMC11841898; doi:10.1371/journal.pone.0318806)
Supplement: Attachment — Submitted filename: response to reviewers.docx [file pone.0318806.s003.docx]

Dear reviewers,

I would like to thank you for your evaluation of my manuscript and for your comments, all of which helped to improve the paper and/or the application.

My responses to individual comments appear in blue font.

Sincerely,

Jean Peccoud

Reviewer #1: Jean Peccoud's manuscript presents a software note on his application for microsatellite genotyping. For years, evolutionary biologists and ecologists working with microsatellite data have expressed frustration with available programs for analyzing capillary electrophoresis data—these programs are often prohibitively expensive, prone to bugs, slow, or have a steep learning curve. The most popular program, GeneMapper, embodies all these issues.

The release of STRyper is game-changing. It is free, fast, resource-efficient, and highly intuitive, enabling us to train students in its use far more quickly than with other programs. Its accuracy is very good, and its size-standard fitting, in my experience, outperforms that of GeneMapper.

While STRyper is currently exclusive to macOS, the author's rationale for this decision is clear. Given its lightweight nature and rapid performance, which does not necessitate a high-performance Mac workstation, I am confident that most labs would gain benefits from it.

This manuscript provides a comprehensive description of the software, leaving no room for further comments. I extend my sincere thanks to the authors for this invaluable contribution to our field.

I thank Dr. Darras for his appreciation of the application and of the paper.

Reviewer #2: This paper presents software programme for Apple Macintosh computers that can help researchers with genotype calling for microsatellites. The programme and its source code are freely available via GitHub. I have tried the programme and it is indeed easy to use with a very powerful GUI. It is great to see that the author created this software as a hobby, and despite the declining use of microsatellites, it will be very useful to evolutionary geneticist. The manuscript describing the software is generally well written and easy to read. However, I do have a number of criticisms, especially regarding the structuring of the text.


Main comments:

My main comment is that the manuscript is structured as a standard experimental paper with a methods section and a results section. This really does not fit the type of paper as it is simply a description of a software programme, so there are not really any methods and results. The paper therefore feels quite repetitive because some parts that logically should be combined are now split into two parts, one in the methods section and one in the results section. I would suggest to forego the methods/results structure in order to present the subjects in a more logical order. I suggest to start with description of the general characteristics of the programme, which is now in the beginning of the results section. The parts on evaluating the application can then be put in the end.

I have restructured the manuscript as suggested, which improved the logic of the text. This required moving several paragraphs, rewriting certain parts, and rearranging figures. I took the opportunity to add a figure showing the genotype list (figure 4). Figure 1 has also been updated to show the most recent version the application and outline the main window components.

Small comments:

l. 37. It is not the motive itself that is mutating, but the number of times that the motive is repeated.

Indeed. I meant “indels affecting the number of microsatellite repeat motives”, as now written.

l. 39-45. I do not see why there are so many details about amplicon sequencing, as this is hardly relevant to the paper.

This was added as a response to previous reviewers. I have removed one sentence. There are only two sentences left on this topic, which I think are useful to readers who may not know the respective advantages of capillary sequencing and of more modern techniques.

l. 54. hence -> therefore

Done.

l. 91. edition -> addition

I meant the “manual correction of DNA ladders”, as now written (lines 88-89)

l. 149. Can you give an explanation why the numbers 10 and 3 are used in this equation?

Yes, this is now explained at lines 229-234. “*The 10/3 coefficient ensures that the score is greatly reduced (often to zero) by a single assignment error, which affects* $\max\left( \frac{{\Delta R}^{2}}{\left| \Delta S \right|} \right)$*. The poor score compels the user to rectify the error. The score is also reduced if certain sizes of the size standard are not assigned to any peak, which increases n_s_–n_p_. A weight of 1/10 was attributed to this component, because such issue generally reflects problems during electrophoresis, which cannot be fixed in the application*.”

l. 162. whose -> that

Fixed.

l. 217-224. I found this description of the method difficult to follow, please revise.

I have rephrased certain parts (lines 292-300): “*Rather than moving bins to match peak positions (which requires maintaining several sets of bins per marker), this approach considers that it is the estimated sizes of peaks (in base pairs), not the position of bins, which should be adjusted. The method thus correct fragment sizes using the formula y = a + bx, where x is the size of a DNA fragment that is estimated by the DNA ladder via the fitted model mentioned earlier, y is the adjusted size, and a and b are constants (hereafter called “offset parameters”). This approach assumes that the effect of varying electrophoresis conditions can be compensated by this linear combination. If there is no correction, a = 0 and b = 1.*”

l. 248-250. I did not understand this.

I have removed that sentence, which was not necessary.

l. 268. Can you specify how the programme works with multiplexes?

The word “panel” referred to multiplexes, as specified at lines 256-258: “*Genotyping requires associating chromatograms with the microsatellite markers that were amplified using fluorescent primers. Markers amplified together by multiplex PCR are regrouped into a “panel” (a term derived from GeneMapper)*”.

l. 275. remove “when the project started”

Apple object-oriented framework no longer require objective C. Swift is the recommended language. Therefore, I think it is useful to specify why objective C was required.

l. 289. Amplicon sequencing can suffer from the same problems with null alleles as gel electrophoresis.

Indeed. I have removed that part.

l. 304. Jelic -> et al.

This has been fixed, though this was how the “PLoS” Endnote style was configured. The style specifies to list the first two authors (without et al.) when there are three or more authors.

l. 348. Rewrite “Most are at a couple of clicks away”

I have rewritten as “*Most can be achieved in a couple of clicks*”.

l. 391. what is meant with “original option”?

It meant that this feature was not found in other applications. This part has been rewritten “*To signal crosstalk to the user, the area underneath an artefactual peak is filled with the color that represents the channel that induced crosstalk (this option can be disabled)*” (lines 176-78).

l. 400. remove “spontaneously”

I have replaced the word with “quickly” because I want to convey the notion that users do not have to switch to a different mode (they must in GeneMapper).

l. 417-423. This part is especially repetitive as most of it is already written in the method section.

This has been reorganized, but I should point out that this was not a repetition. The method section described the way to accommodate variations in electrophoretic position by adjusting offset parameters a and b (which does not change the coordinates of bins). The other section was about modifying a bin set and adjusting the position of bins, as a group or individually. This does not change offset parameters.

l. 447-448. What is meant with “issues in height chromatograms”?

This is a typo, it meant “eight”.

l. 456. So what happened to this marker, was it excluded?

Yes it was, as now specified at line 446.

l. 481-482. This could be explained more clearly.
l. 484-485. I did not understand this.

This part has been simplified. Details that pertain to very specific aspects of the genotype caller were moved to the supplementary material where the genotyping method is described. The text now says “*Rare errors occurred in very specific situations where the length of the alleles differed by only one base pair, such that the shorter peak was considered as the result of adenylation. Only visual comparison with other genotypes showed that adenylation was unlikely. The genotype caller does implement such check by comparing different genotypes called in the same batch, but this check may not always be effective (S1 Text)”*

l. 515. although improvable -> And could be improved upon.

Done.

l. 517-519. My guess is that this would be very difficult as you would need a very large set of high quality training data, where the genotypes have been scored correctly.

Indeed. I now indicate that this perspective is not realistic (lines 500-502): “*Trained artificial intelligence has been proposed for the analysis of chromatograms [**21**], but this approach can only be used on limited set of microsatellite markers.*”.

l. 519. Write “As STRyper, nor any other software, is not immune.

I followed this suggestion, but I replaced “other” by “equivalent”.

Some comments on the software itself (mere suggestions; these do not have to be implemented before the paper can be accepted for publication)

-I noticed that the export function in the file menu was always greyed out, also when the share icon at the bottom of the window was working.

This was because the “Export” menu item can export several types of items: folders, marker panels (and now genotypes). The target of the menu item is either the folder list (left pane) or the marker panel list. If neither is highlighted (i.e., is the first responder of the window), its target cannot be determined. If is therefore greyed out.

However, as the content of the selected folder is always shown (even if empty), the menu now defaults to exporting the selected folder. When there is no selected folder (a case that should only happen after a folder has been deleted), the menu is hidden.

-The binning menu was difficult to find, as it only appears when mousing over the name of the marker. Also, it was not self-evident that the little icon with the three dots was clickable.

Actions involving bins require the marker to be visible as they are performed on the view showing traces. This is why the button to perform these actions is placed on the marker label itself (and is not in a menu from the menu bar or a toolbar button for instance). I can make the button always visible, but it would add visual clutter (as there is one button for each marker). As to whether it is clickable I don’t think there should be much doubt. What would be the point of an icon that appears when a view is hovered, otherwise? The button also and it looks like “action” buttons of other apps, namely the Finder. Note that right-clicking the marker label has the same effect as clicking the button.

As to version 1.3 of the app, adding and editing bins can be achieved by ctrl/right-clicking on the view showing traces within the range of a marker, which pops up a contextual menu.

-The left and right arrows in the chromatogram viewer (“move to next marker”) are not greyed out when there is no next marker to show. It therefore took me some time to discover what it does, as my first clicks did not do anything.

The right arrow is now disabled if the right edge of the last marker is at the left of the right edge of the visible area. An equivalent criterion is used for the left arrow.

-The three icons (import, trash, filter) below the samples panel can be better placed in the window toolbar.

The import button and the trash button have been moved to the toolbar, which received an overhaul. There were many trash buttons to deleted different types of items. Now the toolbar trash button deletes whatever is selected (sample table, folder list, alleles…) and is disabled as appropriate.

A filter button in the toolbar would have to be visually aligned with the list it filters and be immovable (like in Apple Mail). If it is moved far from the table it filters, its action would be unclear, and its removal from the table may lead to problems where an applied filter cannot be removed since the filter button is no longer there. Immovable toolbar items are not supported by the macOS version that the project targets. Moreover, a similar filter button is used to filter genotypes, and it is located below the table it filters. I would rather leave both buttons at their respective location, which is equivalent.

-The plus-icon at the top of the chromatogram viewer, was also quite confusing as it took me a while to figure out that you can only zoom in by dragging the mouse along the ruler on top of the viewer, which is easily missed as the target is very small. There also is no method for zooming out (I tried pressing the alt modifier key but that did not work).

One can now zoom out while clicking with the alt key pressed and to zoom in by a simple click.

Note that one can zoom out by right-clicking or double-clicking the top ruler, via the pinch gesture and via the scroll wheel while holding the alt key. I don’t consider the target that small as the ruler is 14-point tall (as tall as the arrow cursor) and quite large.

The “+” button tooltip indicates that it is used to add a maker.

-It would be nice if there could be an export function to export the called genotypes in a file format that is used by some popular software for population genetic analyses, such as GenAlex, Arlequin, Structure, or –because STRyper is a Mac only programme– GenoDive.

I cannot do that because there is there is no notion of “individual” or “population” in an application that organizes chromatograms. Even the “name” of a sample is often just the well identifier of the sequencer plate. Therefore, I cannot identify chromatograms generated from the same individual.

Also, the application cannot export genotypes with markers taking separate columns, because this would require all samples of a folder to be analyzed at the same marker panel. This would constrain chromatogram management.
